# Interassay Variability and Clinical Implications of Five Different Prostate-specific Antigen Assays

**DOI:** 10.1016/j.euros.2024.03.008

**Published:** 2024-03-21

**Authors:** Basil Kaufmann, Paloma Pellegrino, Laura Zuluaga, Reuben Ben-David, Michael Müntener, Etienne X. Keller, Katharina Spanaus, Arnold von Eckardstein, Michael A. Gorin, Cédric Poyet

**Affiliations:** a Department of Urology, University Hospital Zurich, University of Zurich, Zurich, Switzerland; b Department of Urology, Icahn School of Medicine at Mount Sinai, New York, NY, USA; c Department of Urology, Municipal Hospital of Zurich, Zurich, Switzerland; d Institute for Clinical Chemistry, University Hospital of Zurich, University of Zurich, Zurich, Switzerland

**Keywords:** Prostate-specific antigen assay, Screening, Prostate cancer, Biomarker

## Abstract

**Background and objective:**

Prostate-specific antigen (PSA) remains a critical marker for prostate cancer (PCa) detection and monitoring. Recognising historical variability in PSA assays and the evolution of assay technology and calibration, this study aims to reassess interassay variability using the latest generation of five assays in a contemporary cohort of men undergoing prostate biopsy.

**Methods:**

Five different commercially available PSA assays were tested in a blood sample of 76 men before undergoing a prostate biopsy. Total PSA (tPSA) and free-to-total PSA ratio (%fPSA) were compared across assays, using Roche (Basel, Switzerland) as the benchmark, and correlated with biopsy outcome to analyse the impact on PCa diagnosis. The statistical analysis included Passing-Bablok regression and Bland-Altman plots, with a *p* value threshold of <0.05 for significance.

**Key findings and limitations:**

Among the 76 men, 28 (36.8%) were diagnosed with significant PCa (defined as International Society of Urological Pathology grade ≥2). A high correlation was observed between tPSA and %fPSA values among the different PSA assays tested (*r*^2^ ≥ 0.9). The Passing-Bablok analysis showed that tPSA results varied substantially among the assays, with slopes ranging between 0.78 and 1.04. Compared with the tPSA of Roche, tPSA values were on average 20.7% lower by Beckman (Oststeinbeck, Germany), 15.2% lower by Abbott (Chicago, IL, USA), 6.1% lower by Diasorin (Saluggia, Italy), and 9.6% higher by Brahms (Hennigsdorf, Germany; *p* < 0.001 for all). The %fPSA values by Abbott and Brahms were higher at 15.7% and 10.6%, respectively (*p* < 0.001), while the Beckman and Diasorin values had minimal differences of -0.3% and 2.3%, respectively (*p* > 0.05). The variability across assays would have resulted in discrepancies in both the sensitivity and the specificity for tPSA and %fPSA by at least 14%, depending on the cut-offs applied.

**Conclusions and clinical implications:**

Despite the use of the latest PSA assays, relevant variability of tPSA and %fPSA results can be observed among different assays. There is an urgent need for standardised calibration methods and greater awareness among practitioners concerning interassay variability. Clinicians should acknowledge that clinically relevant thresholds may depend on the specific PSA assay and that ideally the same assay is applied over time for better clinical decision-making.

**Patient summary:**

Prostate-specific antigen (PSA) is a critical marker for prostate cancer (PCa) detection and monitoring. However, significant variations were observed in the results of the latest PSA assays. Thus, standardised calibration methods and greater awareness among practitioners concerning interassay variability are needed.

## Take Home Message

Despite using the latest generation of PSA tests, significant variations in their results were observed. There is urgent need for standardised calibration methods and greater awareness among practitioners concerning inter-assay variability. Clinicians should acknowledge that clinically relevant thresholds may depend on the specific PSA assay and that ideally the same assay is applied over time for better clinical decision-making.

## 1 Introduction

Prostate-specific antigen (PSA) has been used to diagnose and monitor men with prostate cancer (PCa) since its first clinical implementation in the early 1990s [1], [2]. However, despite its widespread use, PSA testing has notable shortcomings, particularly in its specificity and the variability of results from different assays [3], [4], [5]. This inconsistency in PSA levels, a common issue in laboratory practices, underscores the nonuniformity of various manufacturers' assays.

Central to the issue of PSA test inconsistency is the application of universal thresholds for recommending prostate biopsies. The initial threshold for total PSA (tPSA) of 4 µg/l was set in 1986 based on a study by the first assay manufacturer Hybritech Inc. (San Diego, CA, USA) [6]. Although this threshold was later reinforced by large-scale screening studies that contributed to the US Food and Drug Administration’s approval of PSA testing for early PCa detection, it does not account for differences in assay methods, leading to potential misinterpretations and incorrect clinical decisions [7], [8]. Despite the availability of different threshold recommendations from various assay providers [9], these are often ignored in daily clinical practice. The same accounts for clinical research studies, where often not even the test providers are named. This makes replicability and interpretation difficult, and it further leads to wrong risk stratification of patients, with consequences of overdiagnosis and overtreatment or vice versa.

In response to these discrepancies, the World Health Organization (WHO) introduced reference standards for tPSA (code 96/670) and free PSA (fPSA; code 96/668) in 1999, significantly reducing variability and typically lowering tPSA levels by 20–25% [10], [11], [12]. However, even after these recalibrations and adjusting the decision threshold to 3.1 ng/ml, inconsistencies among commercial assays persisted [13]. These are primarily due to the nonequimolar detection of complexed PSA and fPSA, diverse antibody epitope specificities and affinities, and different measurement technologies [14], [15], [16]. Some studies, as of 2006, reported disparities from outdated calibration methods or nonapplication of the specific WHO reference standard [17], [18], [19].

Manufacturers have since made significant efforts to update their analytical platforms and calibration methods [15], [17], [18], [19], [20]. Data from the External Quality Assessment Schemes show an improvement in PSA assay comparability, with tPSA variability reducing from over 20% in 1995 to approximately 7% in 2015 [21]. This progress suggests that current commercial assays may now be harmonised sufficiently for clinical use, considering the analytical performance specifications (APSs) for an acceptable tPSA bias of <±10.6% [15], [22]. The APS sets a benchmark for the maximum allowable deviation from a true or accepted reference value, taking into account its biological variability [15]. However, these findings necessitate careful interpretation, and harmonisation assessments using native clinical samples are recommended [15], [23].

Given this background, our study examines the agreement between tPSA and free-to-total PSA ratio (%fPSA) using the latest and WHO-calibrated PSA assays, some of which were not available at the time of previous studies. Additionally, this study uniquely investigates the implications of PSA discrepancies in the diagnosis of PCa within a contemporary cohort of men presenting with elevated PSA levels. Our findings provide a timely update on an important topic that has only partially been addressed and considered in clinical practice thus far.

## 2 Patients and methods

### 2.1 Setting and participants

The study included 76 men scheduled for a prostate biopsy for suspected PCa, who were willing to participate and donate a blood sample. All the participants underwent a prostate biopsy between February 2019 and July 2020 at the Department of Urology, University Hospital Zurich, while the five PSA assays were performed and analysed at the Institute of Clinical Chemistry, University Hospital Zurich. The exclusion criteria included individuals with a history or current diagnosis of PCa, or those taking 5-alpha reductase inhibitors. A previous negative prostate biopsy was not considered a reason for exclusion. All participants were part of our prospective biopsy outcome study [24]. Clinical data on patients, including factors such as age, race, ethnicity, family history, body mass index, medication history, results of digital rectal examinations, and radiological findings of the prostate, were collected prospectively [24]. The study received approval from the local ethical committee (KEK-Nr. PB-2016-00075), and all participants provided written informed consent in compliance with the study protocol.

### 2.2 Blood samples and analysis

All patient blood samples were obtained just before the scheduled prostate biopsy. Venous blood (10 ml) was collected and centrifuged at 2000 relative centrifugal force. Plasma was separated and aliquoted in 5 × 2 ml fractions and then stored at -80°C until further analysis. All PSA assays were provided by the respective companies, free of charge, for the purpose of the study. The following five PSA assays were used: Roche (Basel, Switzerland), Beckman (Oststeinbeck, Germany), Diasorin (Saluggia, Italy), Brahms (Hennigsdorf, Germany), and Abbott (Chicago, IL, USA). Each assay was carried out according to the manufacturer’s protocol. The institute responsible for conducting the assays was blinded to all clinical and pathological information, with no access to any specific patient-related data or probe information. All samples were measured for tPSA and fPSA using the five assays for direct value comparison within the same patient blood sample. For diagnostic evaluation of each assay, the different threshold limits defined by the manufacturer for tPSA and/or %fPSA were used [18], [25]. The evaluation of %fPSA was conducted exclusively in instances where the tPSA levels were within a “grey zone”, specifically between 4 and 10 ng/ml, in alignment with methodologies described in other literature [26]. The Roche assay was chosen as the reference method, as suggested by Ferraro et al. [9], for a couple of key reasons: Firstly, thresholds for tPSA that guide biopsy decisions were set in a recent study using accurate calibrated models for predicting risk, which relied solely on tPSA measurements from the Roche assay [27]. Secondly, the Roche assay’s credibility is well established due to its thoroughly described assay methodology and proven analytical capabilities [15], [28].

### 2.3 Biopsy and histopathology

All prostate biopsies were carried out as outpatient procedures under general anaesthesia, with patients positioned in lithotomy. To determine the histological grade and location of any tumours, all patients underwent transperineal template saturation and targeted biopsy as described previously [29]. Each biopsy core was assessed individually by a specialised uropathologist. In cases where PCa was diagnosed, the grade was confirmed by a second board-certified pathologist. Clinically significant PCa (csPCa) was defined as International Society of Urological Pathology (ISUP) grade group ≥2, while clinically insignificant PCa (ciPCa) was defined as ISUP grade group 1.

### 2.4 Statistical analysis

For all analyses, the Roche assay served as the reference standard. To measure the strength of agreement and direction of the linear relationship between the different assays and the Roche assay, the Pearson’s correlation coefficient was calculated. The Passing-Bablok regression analysis was used to assess different types of biases. The constant bias is represented by the intercept, and the proportional bias is given by the slope. The 95% confidence intervals (CIs) ascertain whether these values differed significantly from 0 for the intercept and 1 for the slope. If the 95% CI for the intercept included 0, it was inferred that there was no significant constant bias between the methods. Similarly, if the 95% CI for the slope encompassed 1, it was interpreted as an absence of a significant proportional bias between the two methods. Along with the regression analysis, Bland-Altman plots were constructed to show the differences between two compared measurements against the mean of these measurements. The mean difference between the results represents the systematic bias. Paired comparisons of all samples (including tPSA and %fPSA) against the Roche assay were assessed using the Wilcoxon signed-rank test. A two-sided *p* value of <0.05 was considered significant. Data were analysed using the programming language Python version 3.9.13 (Phyton Software Foundation, Wilmington, DE, USA) using Pandas library and Matplotlib version 3.3.3 for data visualisation.

## 3 Results

A total of 76 serum samples were tested for both tPSA and fPSA using five distinct PSA assays. Among these samples, 40 (52.6%) were obtained from patients diagnosed with cancer, encompassing 28 (36.8%) csPCa and 12 (15.8%) ciPCa cases. The remaining 36 samples (47.3%) came from patients with no evidence of PCa. Demographic and histopathological features are depicted in Table 1. The characteristics of the five assays, including the manufacturers' thresholds, are summarised in Table 2.

**Table 1:** Patient characteristics^a^

Variables	Total (*n* = 76)	
Age at biopsy (yr), median (IQR)	64	(59–71)
Prebiopsy tPSA level (ng/ml), median (IQR)	6.3	(4.4–9.8)
Result of prostate biopsy, *n* (%)		
csPCa (ISUP GG 2–5)	28	(36.8)
ISUP grade group 1	12	(15.8)
ISUP grade group 2	9	(11.8)
ISUP grade group 3	7	(9.2)
ISUP grade group 4	10	(13.2)
ISUP grade group 5	2	(2.6)
No cancer, *n* (%)	36	(47.3)
PI-RADS on MRI, *n* (%)		
No lesion or <PI-RADS 3	21	(27.6)
PI-RADS 3	16	(21.1)
PI-RADS 4	24	(31.6)
PI-RADS 5	15	(19.7)
DRE, *n* (%)		
Normal	55	(72.4)
Abnormal	10	(13.2)
Missing	11	(14.5)
Prostate volume (ml), median (IQR)	45.7	(32.3–68.2)
Race, *n* (%)		
White	70	(92.1)
Other	5	(6.6)
Missing	1	(1.3)
Ethnicity, *n* (%)		
Non-Hispanic	75	(98.7)
Hispanic	1	(1.3)
Family history of PCa, *n* (%)		
Positive	18	(23.7)
Negative	58	(76.3)
Prior negative prostate biopsy, *n* (%)		
Yes	14	(18.4)
No	62	(81.6)
BMI (kg/m^2^), median (IQR)	25.9	(32.3–68.2)

BMI = body mass index; csPCa = clinically significant prostate cancer; DRE = digital rectal examination; GG = grade group; IQR = interquartile range; ISUP = International Society of Urological Pathology; MRI = magnetic resonance imaging; PCa = prostate cancer; PI-RADS = Prostate Imaging Reporting and Data System; PSA = prostate-specific antigen; tPSA = total PSA.

a Data are presented as median (IQR) or *n* (%).

**Table 2:** Overview of tested PSA assays

Manufacturer	Analyser	Antibody	Calibrator	Threshold limit total PSA (ng/ml)	Threshold limit %fPSA (%)
Roche	COBAS 8000	Monoclonal	WHO 96/668 WHO 96/670	>4	<25
Beckman	UniCel DxI 800	Monoclonal	WHO 96/668 WHO 96/670	>3.1	<25
Diasorin	Liaison XL	Monoclonal	WHO 96/668 WHO 96/670	>3.2	<10
Brahms	Kryptor Gold	Monoclonal	WHO 96/668 WHO 96/670	>4	<19
Abbott	Architect i1200	Monoclonal	NA	>4	<26

%fPSA = free-to-total PSA ratio; NA = not available; PSA = prostate-specific antigen; WHO = World Health Organization.

**Fig. 1:**
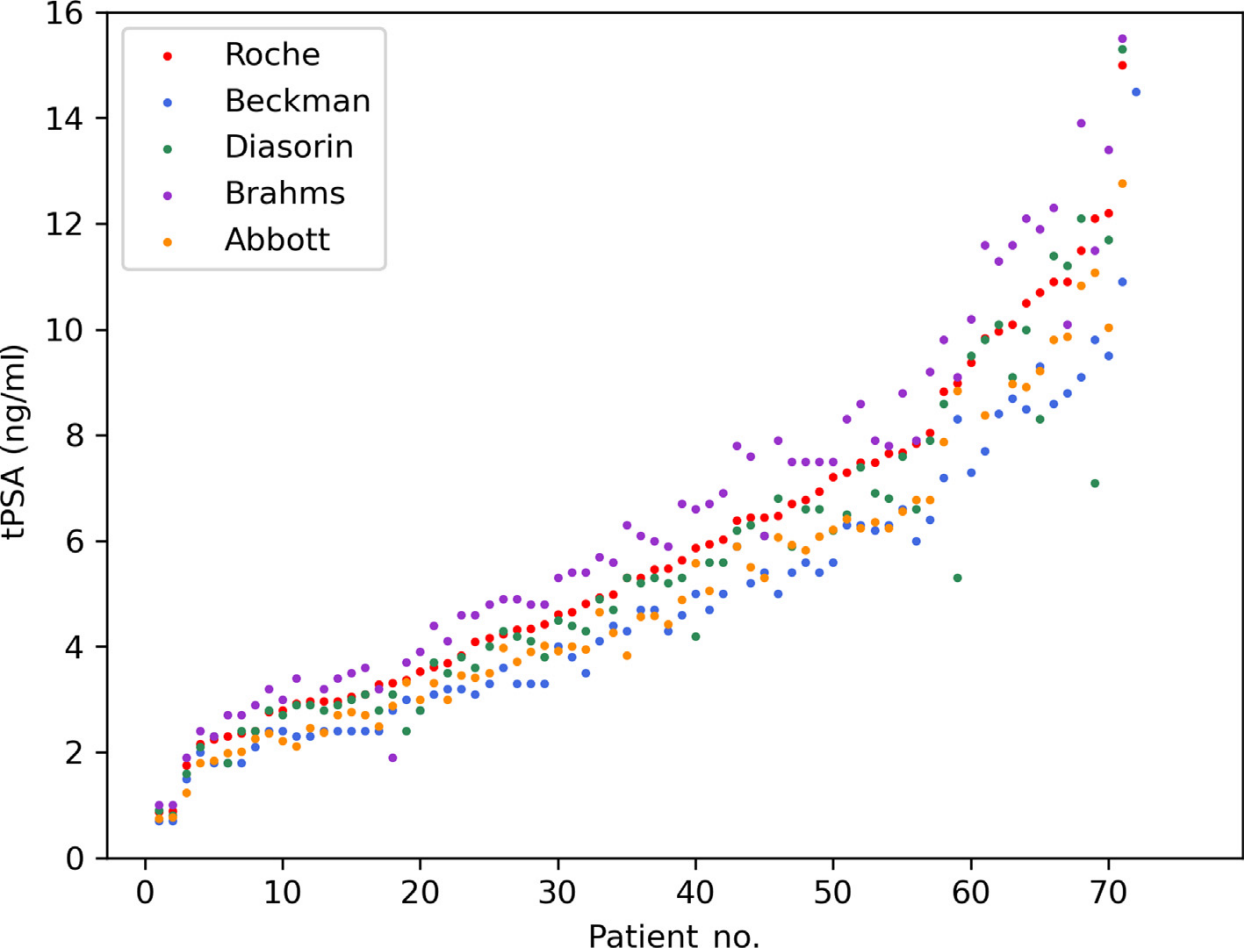
The tPSA values of the five different assays ordered in an ascending fashion by the values of the Roche assay. For better visualisation, outliers >15 ng/ml are not visualised. tPSA = total prostate-specific antigen.

**Fig. 2:**
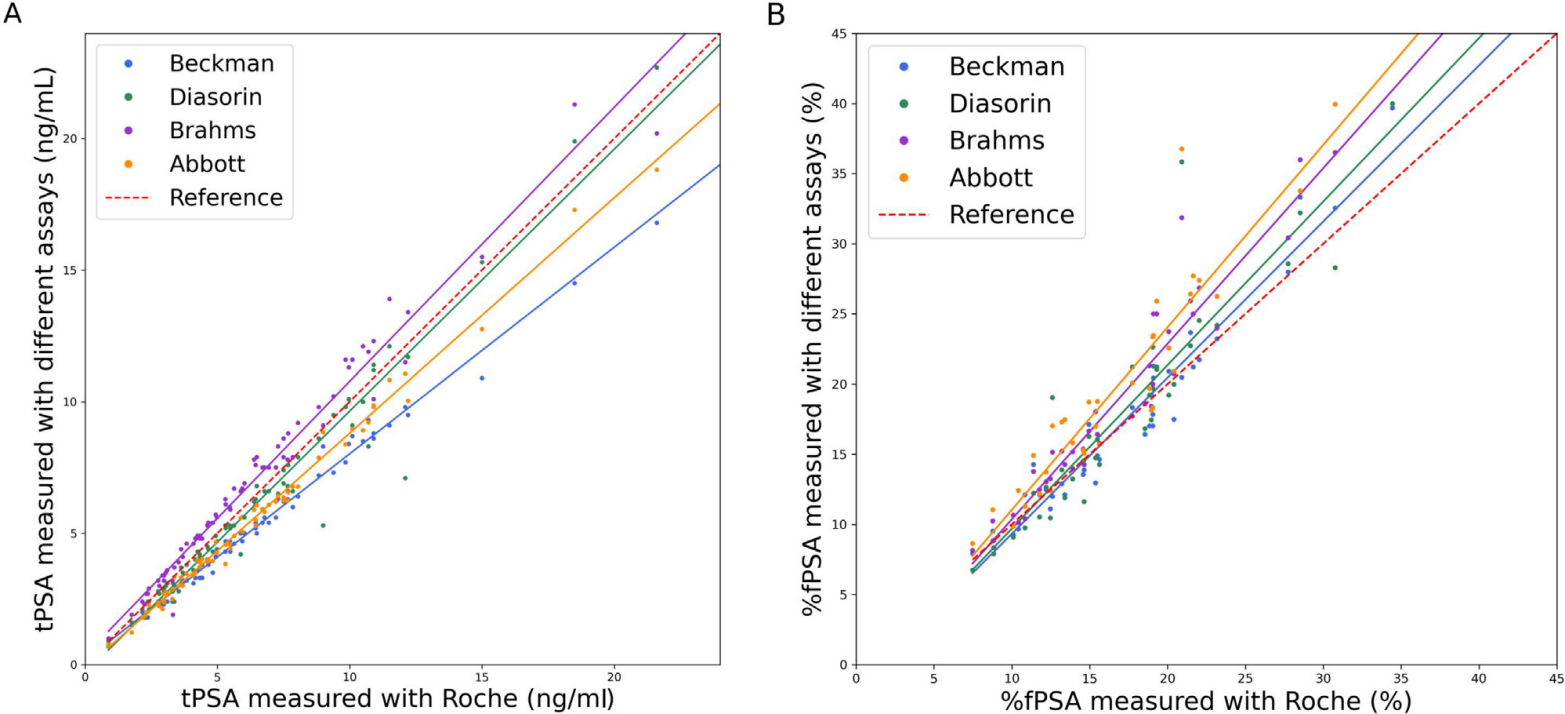
Comparison of tPSA and %fPSA using the Passing-Bablok regression curve analysis. For better visualisation, the *x* axis has been limited to 24 ng/ml, 20 ng/ml, and 45%, respectively. (A) The tPSA results were quite diverse among assays, with Beckman (blue) and Abbott (orange) typically providing lower tPSA readings than Roche (red), while Diasorin (green) and Brahms (purple) generally aligned closely with Roche. (B) The values of %fPSA also demonstrated variation, with the slopes indicating the tendency of overestimation by all assays compared with the Roche (red) assay. %fPSA = free-to-total prostate-specific antigen ratio; tPSA = total prostate-specific antigen.(For interpretation of the references to colour in this figure legend, the reader is referred to the web version of this article.)

**Fig. 3:**
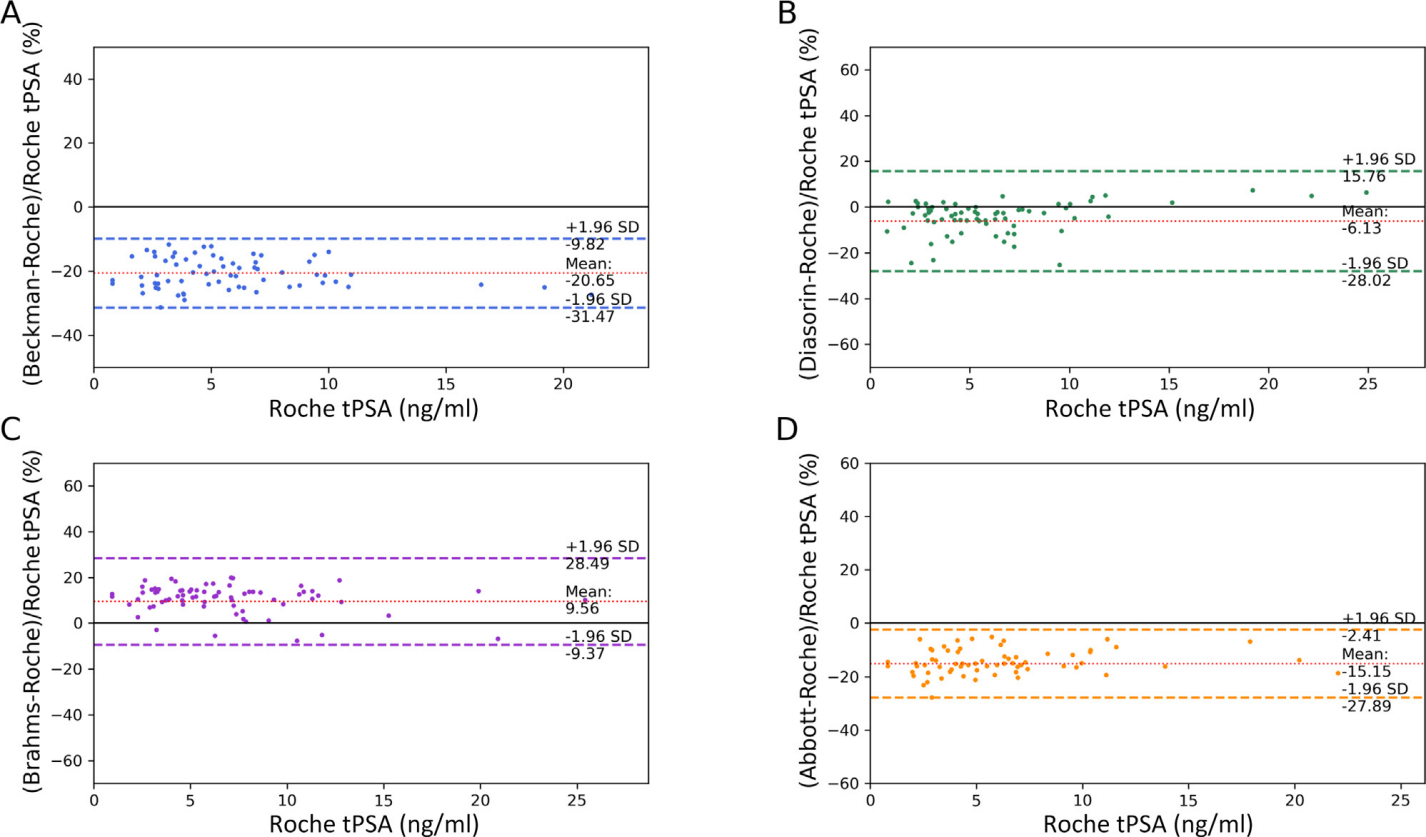
Bland-Altman analysis of tPSA measurements by the five different assays. Beckman underestimated tPSA by 20.7% and Diasorin by 6.13%, Brahms overestimated by 9.6%, and Abbott underestimated by 15.2%, indicating significant discrepancies among assays (*p* < 0.001). SD = standard deviation; tPSA = total prostate-specific antigen.

Figure 1 depicts tPSA results, ordered in an ascending fashion by the values of the Roche assay. The values of the different assays correlated well with the results obtained by Roche, with all Pearson’s correlation coefficients *r*^2^ ≥ 0.97 (Table 3). As shown by the Passing-Bablok regression curve analysis in Figure 2A, the tPSA results were quite diverse among the assays, with slopes ranging between 0.78 and 1.04. Beckman (slope 0.78, 95% CI: 0.77–0.80; intercept 0.17, 95% CI: 0.15–0.19) and Abbott (slope 0.89, 95% CI: 0.88–0.91; intercept -0.14, 95% CI: -0.16 to 0.12) typically provided lower tPSA readings than Roche. On the contrary, Diasorin (slope 1.00, 95% CI: 0.94–1.05; intercept -0.32, 95% CI: -0.37 to 0.26) and Brahms (slope 1.04, 95% CI: 1.00–1.08; intercept 0.36, 95% CI: 0.32–0.40) generally aligned closely with Roche, but Diasorin showed slightly lower and Brahms slightly higher tPSA values. The Bland-Altman analysis, as shown in Figure 3, revealed the extent and consistency of discrepancies. Beckman showed an average underestimation of tPSA values by 20.7%, with limits of agreement ranging from 9.8% to 31.5% (calculated as the mean bias ± 1.96 standard deviation). Diasorin exhibited a modest average underestimation of 6.13%, but with a broad range of agreement from -15.8% to 28%. Brahms showed an average overestimation by 9.6%, with limits of agreement ranging from -28.5% to 9.4%. Lastly, Abbott presented an average underestimation of 15.2%, with limits of agreement from 2.4% to 27.9% (*p* < 0.001 for all assays). All corresponding measurements characteristics are depicted in Table 3.

**Table 3:** Total PSA and %fPSA measurements between the Roche and other assays using the Passing-Bablok regression and Bland-Altman analysis

	Assay	Mean ± SD	Passing-Bablok analysis			Bland-Altman analysis
			Slope (95% CI)	Intercept (95% CI)	Pearson’s correlation coefficient (*r^2^*)	Relative bias (mean ± 1.96SD), %
tPSA (ng/ml)	Beckman	6.45 ± 8.45	0.78 (0.77–0.80)	0.17 (0.15–0.19)	0.99	−20.65 ± 10.82
	Diasorin	7.79 ± 11.17	1.00 (0.94–1.05)	−0.32 (−0.37 to 0.26)	0.97	−6.13 ± 21.89
	Brahms	8.75 ± 10.94	1.04 (1.00–1.08)	0.36 (0.32–0.40)	0.98	9.56 ± 18.93
	Abbott	6.77 ± 8.34	0.89 (0.88–0.91)	−0.14 (−0.16 to 0.12)	0.99	−15.15 ± 12.74
	Roche a	8.03 ± 10.76				
%fPSA (%)	Beckman	0.17 ± 0.07	1.11 (1.03–1.20)	−1.80 (−1.89 to 1.71)	0.97	−0.27 ± 17.74
	Diasorin	0.18 ± 0.08	1.17 (1.01–1.33)	−1.96 (−2.12 to 1.80)	0.92	2.3 ± 27.87
	Brahms	0.19 ± 0.09	1.25 (1.12–1.38)	−2.15 (−2.28 to 2.02)	0.96	10.62 ± 21.03
	Abbott	0.20 ± 0.09	1.30 (1.11–1.49)	−1.96 (−2.15 to 1.77)	0.93	15.74 ± 24.96
	Roche a	0.17 ± 0.06				

CI = confidence interval; %fPSA = free to total prostate-specific antigen ratio; PSA = prostate-specific antigen; SD = standard deviation; tPSA = total prostate-specific antigen.

a Reference assay.

**Fig. 4:**
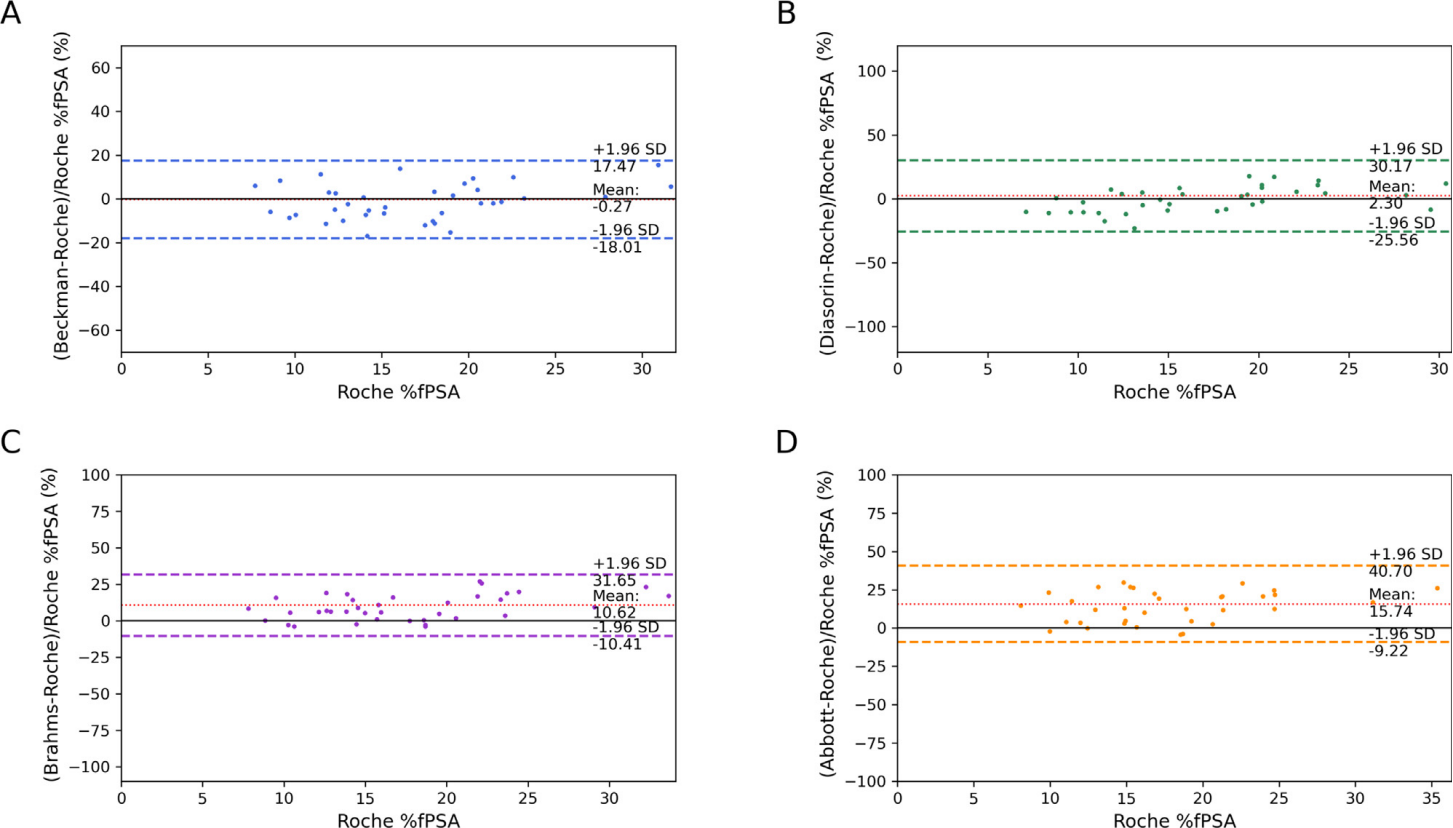
Bland-Altman’s relative bias and precision plots for %fPSA by the five different assays. Beckman and Diasorin showed minimal average differences from Roche (-0.3% and 2.3%, respectively, *p* > 0.5). Brahms and Abbott assays overestimated %fPSA by 10.6% and 15.7%, respectively, indicating significant differences (*p* < 0.001). %fPSA = free-to-total prostate-specific antigen ratio; SD = standard deviation.

Regarding %fPSA, 39 measurements were eligible for analysis since these exhibited tPSA values (determined using the Roche assay) within the range of 4–10 ng/ml. The values of %fPSA also demonstrated variation, as depicted in Figure 2B. The Pearson's correlation coefficients *r^2^* were all over 0.90, indicating a good correlation with the Roche assay. The slope values, as shown in Table 3, indicate the tendency of overestimation by all assays compared with the Roche assay. The Bland-Altman plots in Figure 4 showed that Beckman and Diasorin yielded values quite similar to those of Roche, with minimal average differences of -0.3% and 2.3%, respectively (*p* > 0.5). The corresponding limits of agreement ranged from -18.0% to 17.5% and from -25.6% to 30.2%, respectively. Brahms overestimated the values by 10.6%, with the limits of agreement varying from -10.4% to 31.7%, and Abbott had the highest overestimation of 15.7%, with limits of agreement varying from -9.2% to 40.7% (*p* < 0.001 for both).

To analyse the potential impact of different tPSA and %fPSA measurements from each assay on clinical decisions, we conducted a retrospective analysis of our patient cohort based on their biopsy outcomes. The currently widely adopted, clinically relevant cut-offs were selected and set at 3.1 and 4 ng/ml (each ± 0.2) for tPSA and 25%, 20%, and 15% for %fPSA. Supplementary Table 1 illustrates that the absolute differences in tPSA levels compared with the Roche assay ranged between -0.67 and 0.33 ng/ml at 3.1 ng/ml, and between -0.83 and 0.64 ng/ml at 4 ng/ml. The data also revealed considerable variation in both sensitivity and specificity across different assays and thresholds, with differences reaching up to 14% for sensitivity and 20% for specificity, as shown in Supplementary Table 2. Similarly, for %fPSA, the disparities in sensitivity and specificity were as high as 14% and 26%, respectively, as shown in Supplementary Table 3.

## 4 Discussion

The findings of this study demonstrate considerable inconsistencies among PSA assays, with significant variability in tPSA and %fPSA values among five WHO-calibrated assays from Roche, Beckman, Diasorin, Brahms, and Abbott. The analysis reveals that Beckman and Abbott, compared with Roche, show systematically lower tPSA values by -21% and -15%, respectively, while the Brahms assay demonstrated higher values of +10%. The variability also extended to the %fPSA measurements, with Brahms and Abbott significantly measuring higher percentages by +11% and +16%, respectively, whereas Beckman and Diasorin provided readings similar to the Roche assay. The degree of variability was wide, as reflected by the broad range of limits of agreement in the Bland-Altman analysis. The study further shows that the assay choice could impact the detection of csPCa, impacting both sensitivity and specificity for tPSA and %fPSA by at least 14%, based on the threshold applied.

These inconsistent results underline the on-going issue of discrepancies among PSA assays, despite the introduction of the WHO reference calibration in 1999 [10]. In 2006, Kort et al [18] demonstrated that the Roche (Elycsys) assay, when calibrated with the WHO reference preparation for tPSA, provided tPSA levels nearly identical to those expected for the WHO standard. However, discordance was found when patient samples were used. In 2007, Stephan et al [30] showed that the WHO calibration reduced tPSA results by about 25% compared with the historical Hybritech calibration, recommending a biopsy-triggering threshold lowered from 4 to 3.1 ng/ml. Analysing the manufacturer's thresholds of the assays used in the current study, we found that the threshold values for Roche, Abbott, and Brahms were still to be set at 4 ng/ml, which would not align with this recommendation and could potentially lead to an underestimation of disease. The clinical implications of such interassay variability are significant as the inconsistent results can lead to unnecessary or falsely omitted prostate biopsies. The issue becomes even worse if a patient undergoes serial measurements using different assays.

PCa risk assessment and the indication for biopsy are currently approached in a multivariable manner using risk calculators and nomograms that incorporate factors such as age, multiparametric resonance imaging findings, family history, digital rectal examination, and race [31]. However, PSA continues to serve as a primary marker for PCa. Often, it is the leading factor that prompts the recommendation for a biopsy.

Key areas that require attention include calibration and the number of PSA tests in use. The need for new calibration methods is evident, and existing methods should be either centralised or uniformly implemented across laboratories. A study by Forde et al [32] demonstrated that among nine hospitals in Ireland, only one hospital adhered to the 3.1 ng/ml threshold during recalibration using the WHO 96/670 standard, while all others used the 4 ng/ml threshold. Furthermore, the study revealed that 36 laboratories throughout the country used nine different assays [33]. Howell et al [34] reviewed the main London hospitals and found six different PSA assays in use. These data, regardless of each assay's performance, suggest inadequacy in the current number of available assays. Owing to the diversity of available assays, there is a scenario where patients may be advised to get a biopsy because they had an initial PSA test at one hospital and a subsequent one at another. Often, clinicians do not have details on the particular assay used for determining their patient's PSA. Furthermore, it is advisable to be aware that monitoring patients with PCa should be conducted using the same assay. If a new assay is introduced during the monitoring process, a second measurement within the same sample may be required to compare the results from both assays accurately. Therefore, it is crucial that physicians have a good understanding of method dependencies and, as a consequence, the variability between measuring results as well as their different decision threshold values prior to making clinical judgements [32]. Moreover, the on-going discrepancies between methods despite standardisation warrant the definition of assay-specific rather than universal decision thresholds by both manufacturers and guidelines.

To apply our findings in clinical practice, we recommend the following points: (1) the use of assay-specific thresholds rather than a universal threshold for PSA levels, (2) consistency in assay use to ensure that serial PSA measurements for individual patients are performed using the same assay, and (3) the education of both clinicians and patients about the implications of PSA assay variability, as well as integrating these findings into clinical guidelines.

The present study has several limitations. The sample size is relatively small, and particularly for assessing assay performance, it is not possible to draw definitive conclusions. The cohort was very homogeneous regarding race and ethnicity as compared with other biopsy cohorts [24], and it remains uncertain whether our results would have been altered by a more diverse cohort. For instance, Deng et al [35] found in a Chinese cohort of 163 men a relative bias between Roche and Beckman of <2%, in contrast to our findings of a 21% bias. This discrepancy may be attributable not only to calibration differences (Hybritech vs WHO standard), but also to variations in sample types [36]. The primary strength of this study resides in its simple yet distinctive design, providing clinically relevant data that can benefit every clinician involved in PSA-related matters.

## 5 Conclusions

Despite calibration efforts, significant inconsistencies remain among PSA assays from various manufacturers, leading to considerable variability in tPSA and %fPSA values. These discrepancies can impact the detection of csPCa and lead to unnecessary or falsely omitted prostate biopsies. The current situation underscores the urgent need for standardised calibration methods and greater awareness among practitioners concerning interassay variability. Acknowledging that thresholds may depend on the specific assay used and ensuring the consistent use of the same test for each patient seem vital before making clinical decisions.

  ***Author contributions*:** Basil Kaufmann had full access to all the data in the study and takes responsibility for the integrity of the data and the accuracy of the data analysis.

  *Study concept and design*: Kaufmann, Poyet.

*Acquisition of data*: Kaufmann, Pellegrino.

*Analysis and interpretation of data*: Kaufmann, Poyet.

*Drafting of the manuscript*: Kaufmann, Poyet.

*Critical revision of the manuscript for important intellectual content*: Keller, Müntener, Gorin, Zuluaga, Ben-David, Spanaus.

*Statistical analysis*: Kaufmann.

*Obtaining funding*: Kaufmann.

*Administrative, technical, or material support*: None.

*Supervision*: Poyet, von Eckardstein.

*Other*: None.

  ***Financial disclosures:*** Basil Kaufmann certifies that all conflicts of interest, including specific financial interests and relationships and affiliations relevant to the subject matter or materials discussed in the manuscript (eg, employment/affiliation, grants or funding, consultancies, honoraria, stock ownership or options, expert testimony, royalties, or patents filed, received, or pending), are the following: None.

  ***Funding/Support and role of the sponsor*:** The research was supported by the Swiss Cancer League (Krebsliga Schweiz) and the Swiss Society of Urology.

  ***Acknowledgements*:** We would like to thank Roche (Basel, Switzerland), Beckman (Oststeinbeck, Germany), Diasorin (Saluggia, Italy), Brahms (Hennigsdorf, Germany), and Abbott (Chicago, IL, USA) for providing the assays used in this study.

  ***Data sharing*:** The Python code for statistics and the data used in the analysis are available upon request from the corresponding author.
