# Supplementary material for: Interassay Variability and Clinical Implications of Five Different Prostate-specific Antigen Assays
Source: Eur Urol Open Sci. 2024 Mar 21;63:4–12. doi: 10.1016/j.euros.2024.03.008 (PMC10981008; doi:10.1016/j.euros.2024.03.008)
Supplement: Supplementary data 1 [file mmc1.docx]

| **Assay** | Absolute and Relative Bias at 3.1 ± 0.2 ng/mL | Absolute and Relative Bias at 4 ± 0.2 ng/mL |
| --- | --- | --- |
| ***Beckman*** | -0.67 (-21.6%) | -0.83 (-20.5%) |
| ***Diasorin*** | -0.13 (-4.4%) | -0.23 (-5.6%) |
| ***Brahms*** | 0.33 (10.9%) | 0.64 (15.9%) |
| ***Abbott*** | -0.52 (-17.2%) | -0.57 (-14.25) |
| ***Roche (Reference)*** | - | - |

**Supplementary Table 1:** Absolute and relative bias for tPSA compared to Roche at two different clinical decision levels.
