# Supplementary material for: Interassay Variability and Clinical Implications of Five Different Prostate-specific Antigen Assays
Source: Eur Urol Open Sci. 2024 Mar 21;63:4–12. doi: 10.1016/j.euros.2024.03.008 (PMC10981008; doi:10.1016/j.euros.2024.03.008)
Supplement: Supplementary data 2 [file mmc2.docx]

| **3.1 ng/mL** | | | | | | |  | **4 ng/mL** | | | | | | |
| --- | --- | --- | --- | --- | --- | --- | --- | --- | --- | --- | --- | --- | --- | --- |
| **Assay** | **Sensitivity** | **Specificity** | **TP** | **TN** | **FP** | **FN** |  | **Assay** | **Sensitivity** | **Specificity** | **TP** | **TN** | **FP** | **FN** |
| Roche | 86% | 23% | 24 | 10 | 34 | 4 |  | Roche | 79% | 36% | 22 | 16 | 28 | 6 |
| Beckman | 79% | 30% | 22 | 13 | 31 | 6 |  | Beckman | 71% | 50% | 20 | 22 | 22 | 8 |
| Diasorin | 82% | 27% | 23 | 12 | 32 | 5 |  | Diasorin | 79% | 41% | 22 | 18 | 26 | 6 |
| Brahms | 89% | 16% | 25 | 7 | 37 | 3 |  | Brahms | 79% | 30% | 22 | 13 | 31 | 6 |
| Abbott | 82% | 32% | 23 | 14 | 30 | 5 |  | Abbott | 68% | 48% | 19 | 21 | 23 | 9 |

**Supplementary Table 2:** Diagnostic performance of tPSA according to two different thresholds. TN = true negative;TP = true positive; FP = false positive; FN = false negative
