# Supplementary material for: Interassay Variability and Clinical Implications of Five Different Prostate-specific Antigen Assays
Source: Eur Urol Open Sci. 2024 Mar 21;63:4–12. doi: 10.1016/j.euros.2024.03.008 (PMC10981008; doi:10.1016/j.euros.2024.03.008)
Supplement: Supplementary data 3 [file mmc3.docx]

| **Threshold** | **Test** | **Sensitivity** | **Specificity** | **TP** | **TN** | **FP** | **FN** |
| --- | --- | --- | --- | --- | --- | --- | --- |
| 15% | Beckman | 86% | 94% | 19 | 16 | 1 | 3 |
|  | Diasorin | 91% | 88% | 20 | 15 | 2 | 2 |
|  | Brahms | 100% | 71% | 22 | 12 | 5 | 0 |
|  | Abbott | 100% | 56% | 20 | 9 | 7 | 0 |
| 20% | Beckman | 91% | 93% | 10 | 26 | 2 | 1 |
|  | Diasorin | 91% | 86% | 10 | 24 | 4 | 1 |
|  | Brahms | 100% | 86% | 11 | 24 | 4 | 0 |
|  | Abbott | 100% | 85% | 10 | 22 | 4 | 0 |
| 25% | Beckman | 100% | 100% | 4 | 35 | 0 | 0 |
|  | Diasorin | 100% | 94% | 4 | 33 | 2 | 0 |
|  | Brahms | 100% | 83% | 4 | 29 | 6 | 0 |
|  | Abbott | 100% | 82% | 3 | 27 | 6 | 0 |

**Supplementary Table 3:** Diagnostic performance of %fPSA according to three different thresholds. TN = true negative;TP = true positive; FP = false positive; FN = false negative
